# IgG4-related disease presenting with retroperitoneal fibrosis and hypertrophic spinal pachymeningitis: a rare case report and literature review

**DOI:** 10.3389/fimmu.2025.1619756

**Published:** 2025-08-29

**Authors:** Jianchun Wang, Feifan Xu, Hongzhou Duan, Juan Zhao, Haoze Zhang, Jingru Ren, Zhenyu Niu, Feng Gao, Siwei Chen, Ran Liu

**Affiliations:** ^1^ Department of Neurology, Peking University First Hospital, Beijing, China; ^2^ Department of Neurosurgery, Peking University First Hospital, Beijing, China; ^3^ Department of Rheumatology and Clinical Immunology, Peking University First Hospital, Beijing, China

**Keywords:** IgG4-related disease, retroperitoneal fibrosis, hypertrophic spinal pachymeningitis, immunosuppressive therapy, surgical decompression

## Abstract

**Background:**

Hypertrophic spinal pachymeningitis (HSP) and retroperitoneal fibrosis (RPF) are rare inflammatory disorders, often associated with immunoglobulin G4-related disease (IgG4-RD). This case underscores the diagnostic intricacies and therapeutic challenges in a patient with overlapping neurological and systemic manifestations.

**Case presentation:**

A 34-year-old female presented with concurrent RPF and HSP leading to urinary tract obstruction and progressive spinal cord compression. The patient initially presented with lower limb weakness and urinary dysfunction, followed by acute paraparesis after a fall. Despite normal serum IgG4 levels, a pathological examination of the dural biopsy confirmed the diagnosis of IgG4-RD. Following a multidisciplinary treatment approach that included surgical decompression, glucocorticoids, immunomodulators and antifibrotic therapy, the patient achieved a favorable clinical outcome.

**Literature review:**

A systematic review of 22 cases involving patients with HSP revealed that key manifestations included localized pain, motor deficits, sensory abnormalities and autonomic dysfunction. The majority of patients (82%, 18/22) exhibited isolated HSP, with a predominance of thoracic spine involvement. Pathological examination demonstrated lymphoplasmacytic infiltration in all cases (100%, 22/22), with 95% (19/20) meeting the criteria for IgG4-positive plasma cells; storiform fibrosis and obliterative phlebitis were observed in 56% (5/9) of cases. Treatment strategies primarily involved surgical decompression (95%, 21/22) and glucocorticoids (95%, 21/22), with 52% (11/21) receiving additional immunosuppressive agents. Clinical outcomes showed complete neurological recovery in 19% (4/21), partial recovery in 71% (15/21). Recurrence was documented in 17% (3/18) of patients with available follow-up data.

**Conclusion:**

This rare case underscores the importance of integrating clinical, radiological, and histopathological findings to diagnose HSP and RPF, particularly in the context of IgG4-RD. Early multidisciplinary management is critical to improving outcomes.

## Introduction

IgG4-related disease (IgG4-RD) is a rare systemic fibro-inflammatory disorder characterized by fibrous tissue proliferation and inflammatory cell infiltration, which can affect multiple organs, including the pancreas, salivary glands, lacrimal glands, retroperitoneum, kidneys, lungs and thyroid gland (1). The core pathological features of this disease include lymphoplasmacytic infiltration, storiform fibrosis, occlusive phlebitis and an abundance of IgG4-positive (IgG4+) plasma cells (2). This disease was previously misdiagnosed as isolated single-organ diseases (such as autoimmune pancreatitis and Kuttner tumor), until its recognition as a systemic disease in 2003 (1).

Retroperitoneal fibrosis (RPF) is also a rare disorder characterized by the encasement of the abdominal aorta, ureters, and adjacent abdominal organs in dense fibrotic tissue. This encapsulation leads to clinical manifestations such as obstructive uropathy, vascular compression and systemic symptoms. The fibrotic process is believed to be associated with the aberrant expression of cytokines and growth factors that promote fibroblast activation and extracellular matrix deposition. Though the etiology of RPF remains incompletely understood, a subset of cases is strongly associated with IgG4-RD (3, 4). IgG4-related RPF (IgG4-RPF) is considered a secondary form of RPF, showing significant differences in clinical features compared to idiopathic RPF. Patients with IgG4-RPF typically exhibit elevated serum IgG4 levels, increased eosinophil counts, and higher IgE levels (5).

Hypertrophic pachymeningitis (HP), characterized by thickening of the dura mater or spinal meninges, can result in localized pain and neurological deficits. It may occur idiopathically or secondary to various underlying conditions, including IgG4-RD and anti-neutrophil cytoplasmic antibody (ANCA) associated vasculitis (6). Meningeal involvement is a common manifestation of IgG4-RD; however, IgG4-related hypertrophic spinal pachymeningitis (IgG4-HSP) is exceedingly rare. This article presents an extremely rare case of IgG4-HSP coexisting with RPF.

## Case presentation

### Clinical history

**Figure 1:**
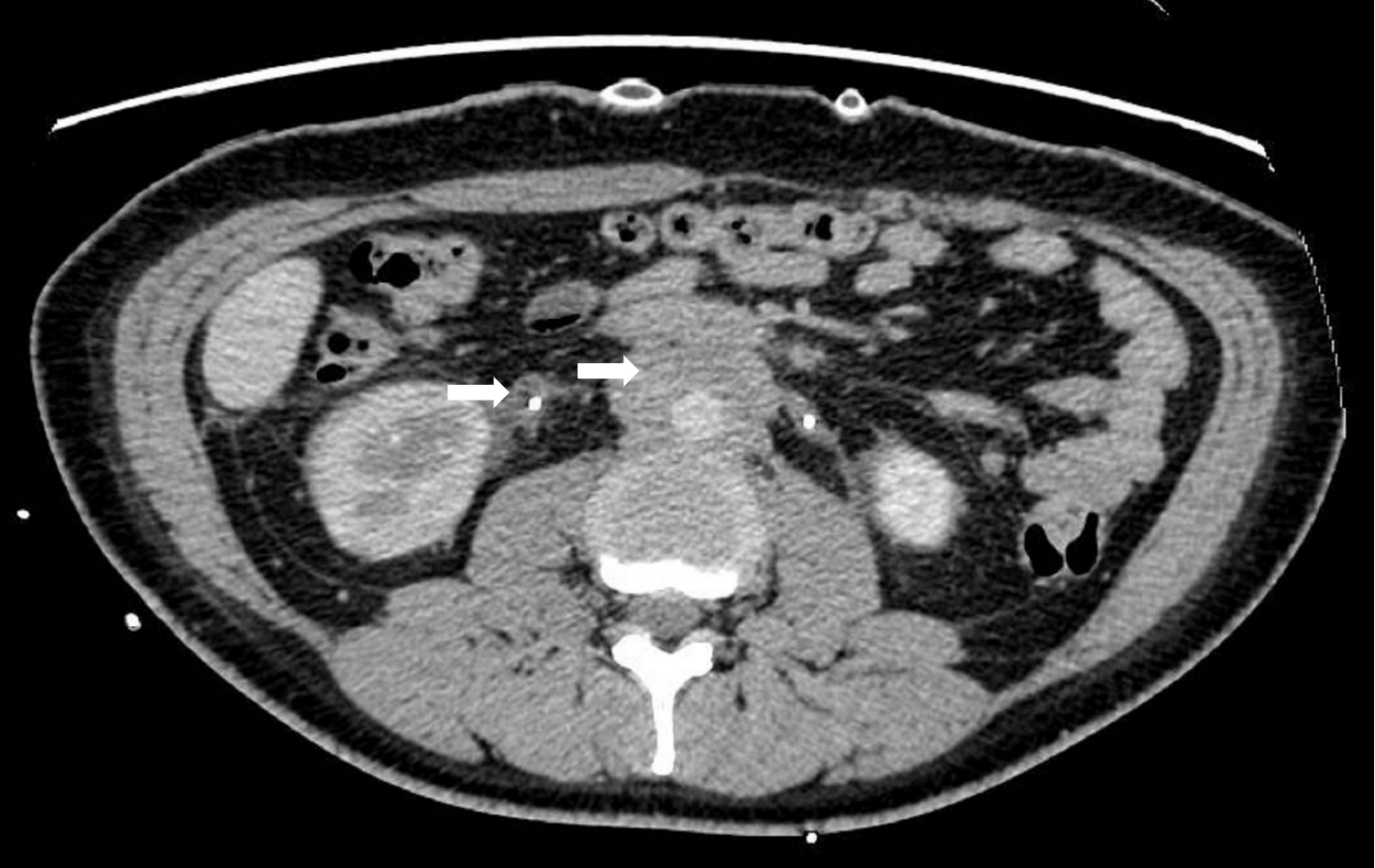
Computed tomography findings of retroperitoneal fibrosis. The abdominal computed tomography scan showed soft tissue densities around the abdominal aorta and ureter.

A 34-year-old female was admitted to hospital six months prior due to intermittent lower back pain and urinary tract obstruction. She underwent bilateral ureteral stent implantation and bilateral nephrostomy. Abdominal computed tomography (CT) scans revealed multiple soft tissue densities in the retroperitoneal region (**Figure 1**, arrows). Five months ago, she underwent a retroperitoneal space puncture and pathological examination of the retroperitoneal mass indicated proliferative fibrous tissue with infiltration of lymphocytes and plasma cells. Immunohistochemical staining showed CD3(++), CD20(+), CD138(++), with scattered IgG(+) and IgG4(+) plasma cells. The serum IgG4 was normal. As she did not meet the diagnostic criteria for confirmed IgG4-RD, she was diagnosed with idiopathic RPF and subsequently initiated on rituximab therapy.

2 weeks ago, she fell over in bathroom and suffered injuries to the right shoulder and mandible. X-ray imaging revealed no significant fractures. The subsequent morning, she exhibited bilateral weakness of the lower limbs, hypoesthesia, and an inability to ambulate, accompanied by centralized localized spinal pain at the scapular level. Due to the progressive exacerbation of symptoms, she was admitted to our neurology department.

### Physical examination

Bilateral nephrostomies were observed in the abdominal region.

Sensory examination revealed hypoalgesia and hypopallesthesia below the bilateral T4 level, with impaired deep sensation and an absence of vibratory sense below both knee joints.

In terms of motor function, proximal muscle strength was assessed at 0/5 and distal muscle strength at 2/5 in both lower limbs. Clasp-knife hypertonia was noted in both lower limbs.

Reflex examination indicated the absence of bilateral abdominal wall reflexes. Bilateral knee tendon reflexes were symmetrically hyperactive. Additionally, bilateral patellar and ankle clonus were elicitable. The Rossolimo, Babinski, Chaddock, and Gordon signs were all positive bilaterally.

### Diagnostic workup

Laboratory investigations showed normal serum IgG4 levels (0.9 mg/dL), with the erythrocyte sedimentation rate (ESR) and serum levels of IgG, IgA, and IgM all within normal limits. The antinuclear antibody (ANA) panel, ANCA antibody, vascular endothelial growth factor (VEGF), and tumor markers were all negative. Additionally, screening tests for human immunodeficiency virus (HIV), syphilis, and hepatitis viruses also yielded negative results.

**Figure 2:**
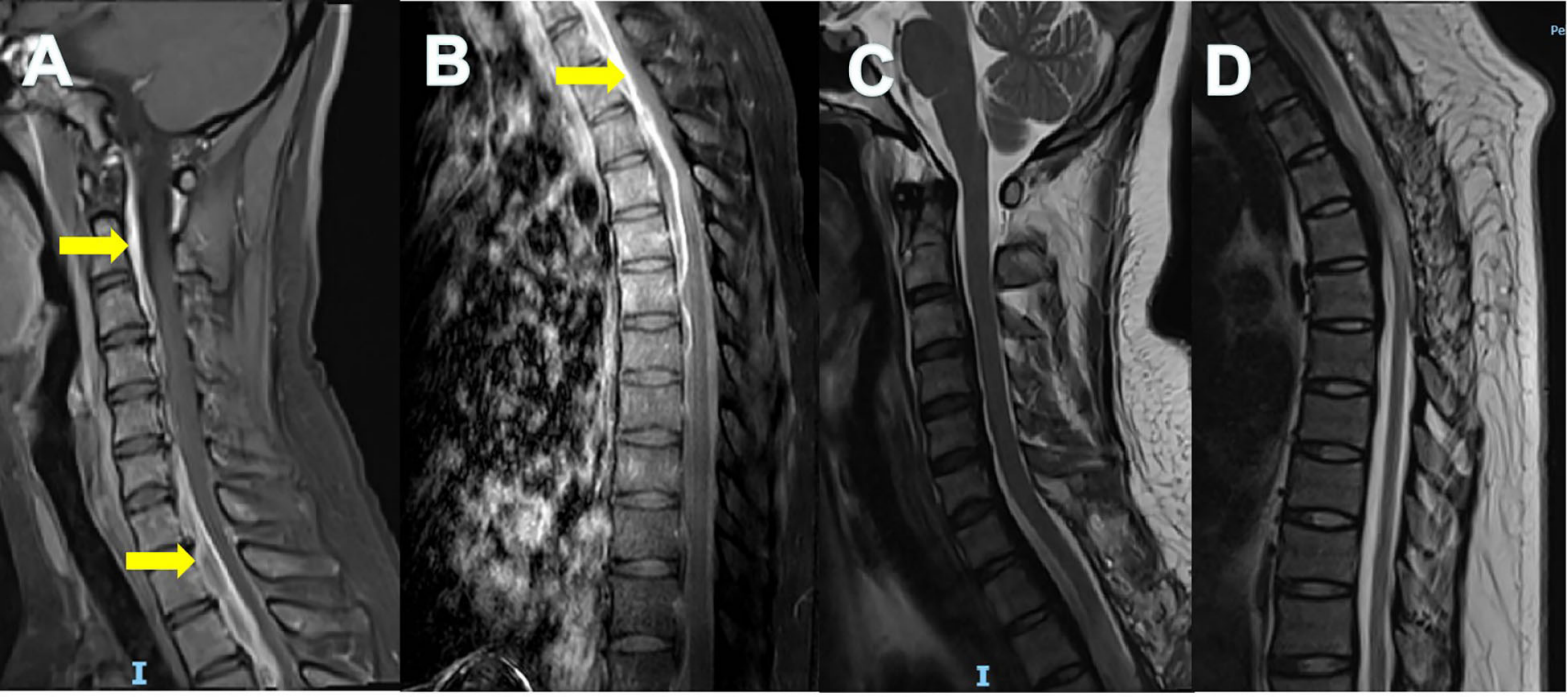
Magnetic resonance imaging findings of spinal dura mater. Enhanced MRI of cervical **(A)** and thoracic **(B)** spine showed thickening and uneven enhancement of the spinal dura mater with spinal cord compression (yellow arrows). The thickened and strengthened dura presented as the “Sandwich” sign. The unenhanced part in the middle was considered to have more fiber or collagen components. MRI of cervical **(C)** and thoracic **(D)** spine at 8-month follow-up showed reduced spinal cord compression.

Spinal magnetic resonance imaging (MRI) revealed abnormal signals at the anterior edge of the dural sac and the posterior longitudinal ligament area with spinal compression observed at the C3-T5 level (refer to **Figures 2A, B**, indicated by the yellow arrow).

**Figure 3:**
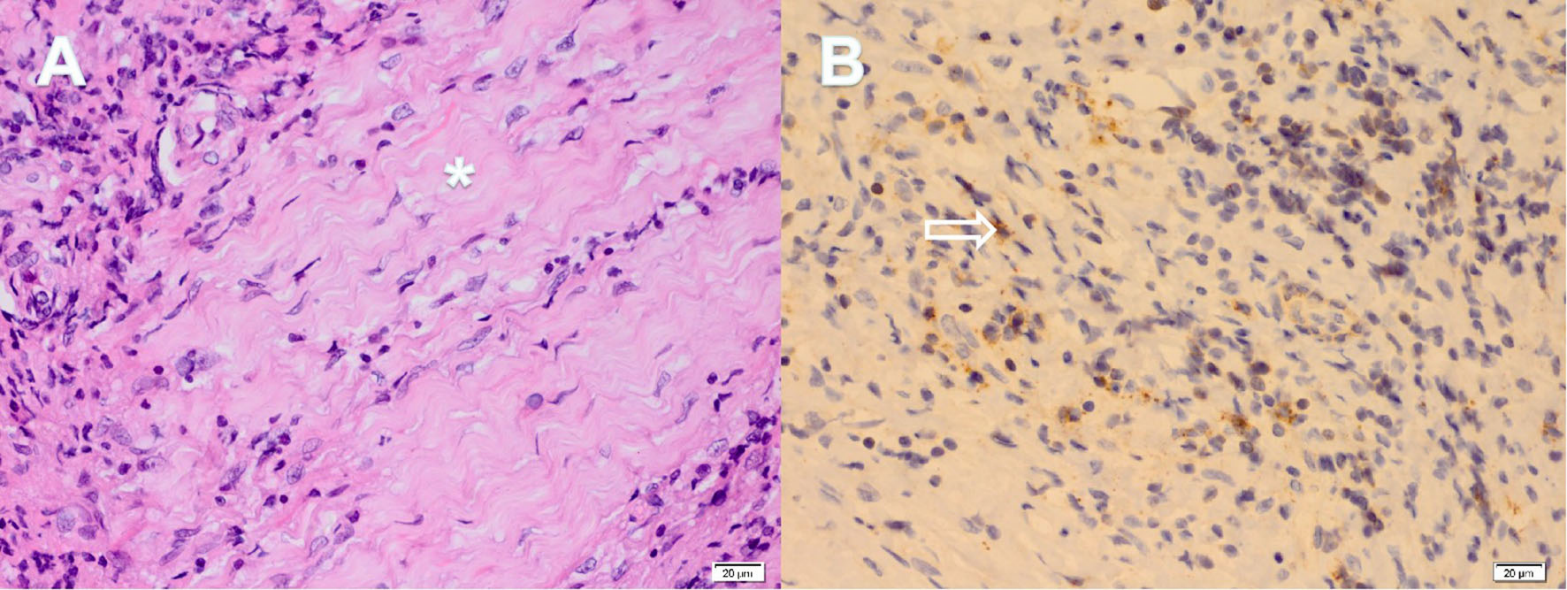
Partial pathological results of dural biopsy. The pathology of the dural biopsy revealed the following: Hematoxylin-Eosin staining **(A)** showed the storiform fibrosis of tissue (asterisk), accompanied by lymphocyte, eosinophil and plasma cell infiltration; Immunohistochemical staining **(B)** demonstrated IgG4+ plasma cells (hollow arrow), and the number of which was greater than 10 per high powered field.

Pathological examination of the dural biopsy demonstrated storiform fibrosis (see **Figure 3A**, marked by an asterisk), accompanied by infiltration of lymphocytes, eosinophils, and plasma cells. Although IgG4+ plasma cells were sparse, more than 10 cells per high-power field (HPF) were identified only in one focus (see **Figure 3B**, indicated by the hollow arrow). However, the ratio of IgG4+/IgG+ in biopsied dural tissue was lower than 40% in the overall pathological tissue examined.

Based on these findings, RPF and HSP were confirmed, and she was diagnosed with probable IgG4-RD in accordance with the 2020 revised comprehensive diagnostic criteria for IgG4-RD (7).

### Therapy and follow-up

She underwent surgical decompressive laminectomy and received high-dose glucocorticoids (methylprednisolone 240 mg/day) for 4 days, followed by a regimen of oral prednisone in combination with azathioprine (AZA) and tamoxifen therapy.

At the 3-month follow-up, she exhibited significant improvement in paraesthesia, and the muscle strength in her lower limbs increased to grade 4. She underwent the removal of bilateral nephrostomy tubes, and prednisone was gradually tapered.

By the 8-month follow-up, her motor strength in the lower limbs had returned to normal, but hyperreflexia and Babinski signs persisted. Imaging studies indicated a reduction in spinal cord compression (**Figures 2C, D**) and the retroperitoneal mass had also regressed.

## Literature review on 22 cases of IgG4-HSP

IgG4-RD are indeed a rare immune-mediated disorder that can affect multiple organ systems, including the nervous system. The involvement of the nervous system in IgG4-RD is primarily seen in the meninges and the pituitary gland. However, involvement of the spinal dura is rarely reported, and the combination of RPF and HSP is even more rare. A literature search was conducted using the PubMed and Embase databases with the keywords “IgG4” and “hypertrophic spinal pachymeningitis”, which identified 22 relevant case reports (as shown in **Table 1**).

**Table 1:** Clinical data of the 22 cases of IgG4-related hypertrophic spinal pachymeningitis.

No.	Age/Sex	Clinical manifestation	Duration	Lesion site on spinal MRI	Involvement of other systems	Serum IgG4	Pathological features of spinal dura mater				Treatment	Outcome and follow-up
							Infiltration of lymphocytes and plasma cells	Storiform fibrosis or Obliterative phlebitis	The numbers of IgG4+ plasma cells >10/HP	The ratio of IgG4+/IgG+ plasma cells >40%		
1 (8)	37/M	Motor and sensory dysfunction of bilateral lower limbs	2 weeks	T5-T10	Sialisterium	Not mentioned	Yes	Not mentioned	Yes	Yes	Decompression surgery	Partial recovery
2 (9)	55/M	Motor and sensory dysfunction of bilateral lower limbs	2 weeks	T2-T3	No	Elevated (512 mg/dL)	Yes	No	No	Not mentioned	Decompression surgery and GC	Partial recovery
3 (10)	49/F	Right upper back pain	1 month	T1-T4	No	Not mentioned	Yes	Yes	Yes	Yes	Decompression surgery and GC	Not mentioned
4 (11)	79/F	Back pain, motor and sensory dysfunction of bilateral lower limbs, bladder dysfunction	2 weeks	C6/7-L1/2	No	Elevated (value unknown)	Yes	Yes	Yes	Yes	Decompression surgery, GC and RTX	Recovered partially but died of infection
5 (12)	62/M	Cavernous sinus syndrome, right retro-orbital headache, motor and sensory dysfunction of bilateral lower limbs	3 months	C4-T1	Right orbit and cavernous sinus	Normal	Yes	Not mentioned	Yes	Not mentioned	Decompression surgery, GC and RTX	Partial recovery
6 (13)	48/F	Lower back pain, neurogenic claudication, and right lower extremity radiculopathy	2 months	L2-L3	No	Normal	Yes	Not mentioned	Yes	Yes	Decompression surgery and GC	Partial recovery and no recurrence
7 (14)	50/M	Upper back pain, sensory dysfunction of bilateral lower limbs	3 months	C7-T5	No	Normal	Yes	No	Yes	Yes	Decompression surgery, GC and RTX	Partial recovery and no recurrence
8 (15)	58/F	Back pain, motor and sensory dysfunction of bilateral lower limbs, bowel and bladder dysfunction	6 weeks	T2-T7	No	Elevated (327 mg/dL)	Yes	No	Yes	Yes	Decompression surgery, GC and CTX	Recovered partially but died of pulmonary fungal infection
9 (16)	24/M	Back pain, motor and sensory dysfunction of bilateral lower limbs	4 years	C7ars	No	Elevated (220 mg/dL)	Yes	Yes	Yes	Yes	Decompression surgery and GC	Complete recovery and no recurrence
10 (17)	37/F	Neck and back pain, motor and sensory dysfunction of both hands	Not mentioned	C2-T2/3	Chondrite and sialisterium	Not mentioned	Yes	Not mentioned	Yes	Not mentioned	Decompression surgery, GC and MTX	Recovered completely but recurred 2 years later
11 (18)	56/M	Neck pain, gait disorder and cranial nerve palsy	3 months	C2-C4	No	Not mentioned	Yes	Not mentioned	Yes	Yes	Decompression surgery and GC	Partial recovery
12 (19)	34/M	Back pain, motor and sensory dysfunction of bilateral lower limbs	3 months	T3-T5	No	Normal	Yes	Yes	Yes	Not mentioned	Decompression surgery, GC and AZA	Partial recovery and no recurrence
13 (20)	35/F	Pain in the neck and right shoulder, motor and sensory dysfunction of right arm	6 months	C6-T2	No	Normal	Yes	Not mentioned	Yes	Yes	Decompression surgery and GC	Recovered completely but recurred 1 year later
14 (21)	61/F	Back pain, motor and sensory dysfunction of bilateral lower limbs, bowel dysfunction	1 year	T7-T11	No	Elevated (441 mg/dL)	Yes	Yes	Yes	Not mentioned	Decompression surgery and GC	No recovery
15 (22)	49/F	Back pain, motor dysfunction of bilateral lower limbs, bladder dysfunction	1 month	T1-T9	No	Elevated (150 mg/dL)	Yes	Not mentioned	Yes	Yes	Decompression surgery, GC and MTX	Partial recovery and no recurrence
16 (22)	72/F	Back pain, motor and sensory dysfunction of bilateral lower limbs	4 months	C7-T8	No	Normal	Yes	Not mentioned	Yes	Yes	Decompression surgery and GC	Partial recovery and no recurrence
17 (23)	69/F	Back pain	4 months	T1-T4	No	Elevated (139 mg/dL)	Yes	Not mentioned	Not mentioned	Not mentioned	Decompression surgery, GC + RTX →MTX + MMF	Partial recovery and no recurrence
18 (23)	67/M	Bilateral thighs pain	6 months	L2-L3	No	Normal	Yes	Not mentioned	Yes	Not mentioned	Decompression surgery, GC and AZA	Partial recovery and no recurrence
19 (24)	27/M	Motor and sensory dysfunction of bilateral lower limbs	3 weeks	T2-T4	No	Normal	Yes	Not mentioned	Yes	Yes	Decompression surgery and GC	Recovered completely but recurred 4 years later
20 (25)	65/F	Pain in the left arm, sensory dysfunction of bilateral lower limbs, gait ataxia	4 months	T1-T8	No	Normal	Yes	Not mentioned	Yes	Yes	Decompression surgery, GC and RTX	Partial recovery and no recurrence
21 (26)	45/M	Neck pain, motor and sensory dysfunction of right arm	2 months	C2-C5	No	Not mentioned	Yes	No	Yes	Yes	Decompression surgery, GC and RTX	Partial recovery and no recurrence
22 (27)	70/M	Motor and sensory dysfunction of bilateral lower limbs, bowel and bladder dysfunction	6 weeks	T9-L1	No	Elevated (318 mg/dL)	Not mentioned				GC	Partial recovery and no recurrence

GC, glucocorticoid; RTX, rituximab; MTX, methotrexate; AZA, azathioprine; CTX, cyclophosphamide; MMF, mycophenolate mofetil.

This comprehensive analysis of 22 cases elucidates the multidimensional characteristics of IgG4-HSP. The patient cohort predominantly comprises youth to elderly individuals (mean age: 52 ± 15 years, range: 27–79 years), with an equal distribution of male and female subjects. The primary clinical manifestations include localized pain (82%, 18/22 cases), motor deficits (82%, 18/22 cases), and sensory abnormalities (77%, 17/22 cases), with 23% (5/22 cases) experiencing bowel or bladder dysfunction. Elevated serum IgG4 levels (>135 mg/dL) are detected in 47% (8/17 tested cases), while 53% (9/17 cases) exhibit normal levels. Thoracic spinal involvement is most prevalent on spinal MRI (82%, 18/22 cases), followed by cervical segments (41%, 9/22 cases) and lumbar segment (14%, 3/22 cases). It is worth noting that, the majority of patients present with isolated HSP, while only 18% (4/22 cases) showing multi-organ involvement, including sialisterium, orbit, cavernous sinus and chondrite.

Pathological confirmation through dural biopsy reveals lymphoplasmacytic infiltration in all cases (22/22), with 95% (19/20 tested cases) meeting the criterion of having more than 10 IgG4+ plasma cells per HPF or an IgG4+/IgG+ plasma cell ratio exceeding 40%. Only nine instances documented the presence of pathological changes, specifically storiform fibrosis or obliterative phlebitis, with 56% (5/9 cases) exhibiting such changes.

Regarding therapeutic interventions, 95% (21/22 cases) of patients underwent surgical decompression due to spinal compression. Glucocorticoid (GC) therapy was administered to 95% (21/22) of the patients, and 52% (11/21) of these patients received additional immunosuppressive agents, including rituximab (55%, 6/11 cases), methotrexate (27%, 3/11 cases), azathioprine (18%, 2/11 cases), cyclophosphamide (9%, 1/11 cases) and mycophenolate mofetil (9%, 1/11 cases).

Among the 21 patients with documented outcomes (excluding one case with an unspecified prognosis), 19% (4/21 cases) achieved complete neurological recovery following treatment, while 71% (15/21 cases) exhibited partial recovery. Unfortunately, 10% (2/21 cases) succumbed to severe infectious complications. Of the 18 patients with available follow-up data, only 17% (3/21 cases) experienced disease recurrence.

## Discussion

IgG4-RD is a multi-organ, immune-mediated inflammatory disorder with pathogenic mechanisms that remain only partially elucidated. Current evidence suggests that dysregulation of both innate and adaptive immunity plays a central role in the pathogenesis of IgG4-RD. Within the adaptive immune system, multiple T-cell and B-cell subsets are implicated. For instance, aberrant activation of T helper 2 cells (Th2), regulatory T lymphocytes (Tregs), and CD4+ cytotoxic T lymphocytes (CTLs) leads to the production of various cytokines that drive IgG4 production and tissue fibrosis (28). Simultaneously, IgG4 antibodies generated by B cells may bind to antigens and activate downstream immune responses. Nevertheless, some researchers suggest that IgG4 may exert an anti-inflammatory regulatory role through low-affinity antigen binding, rather than acting as a direct pathogenic agent (29). Additionally, genetic predisposition is implicated in the susceptibility to IgG4-RD, with studies associating specific human leukocyte antigen (HLA) and non-HLA genotypes with disease risk or relapse (30).

IgG4-RD has the potential to impact nearly all organs and tissues within the body. Organs characterized by high vascular density, such as the pancreas and kidneys, along with lymphoid-rich regions, including lymph nodes and salivary glands, are particularly susceptible to its effects (2). Certain lymphocyte distributions significantly influence the pattern of organ involvement. For example, CD4+SLAMF7+ CTLs are predominantly infiltrated in the salivary glands, lacrimal glands, and pancreas, potentially resulting in tissue fibrosis and damage (31). The clinical phenotype of RPF/aortitis is primarily characterized by the presence of CX3CR1+ CTLs as the key immune cell subset, whereas Mikulicz disease with systemic involvement is predominantly associated with Th2 (32).

Based on clinical, radiological, and pathological characteristics, as well as responses to immunosuppressive therapies, IgG4-RD can be classified into proliferative and fibrotic phenotypes (2). The pathological characteristics of both phenotypes of IgG4-RD are predominantly characterized by infiltration of IgG4+ plasma cells and the formation of fibrotic tissue. The proliferative phenotype is distinguished by extensive infiltration of lymphoplasmacytic cells, the formation of germinal centers, and significant proliferation of IgG4+ plasma cells, accompanied by relatively mild storiform fibrosis. Unlike the proliferative phenotype, fibrotic IgG4-RD predominantly affects extraglandular tissues, including the retroperitoneum, mediastinum, and meninges, and is characterized by less pronounced elevations in serum IgG4 levels (2). In affected organs, fibrotic features predominate, such as atypical tertiary lymphoid structures, a sparse presence of immune cells, and a reduced number of IgG4+ plasma cells (33). The relationship between fibrotic IgG4-RD and proliferative IgG4-RD remains ambiguous; it is uncertain whether these conditions represent a continuum or distinct disease phenotypes within specific organs. Research focusing on pancreatic involvement in IgG4-RD indicates that the initial inflammatory infiltration by lymphocytes is progressively supplanted by extensive collagen deposition. This observation supports the notion of a temporal progression from a proliferative to a terminal fibrotic stage in IgG4-RD (2). Nonetheless, it remains to be determined whether these concepts are universally applicable across all organs affected by IgG4-RD, or whether the progression may be accelerated in particular sites, such as the retroperitoneum, thyroid, and meninges.

The diagnosis and management of IgG4-RD remain challenging. A significant presence of IgG4+ plasma cells in the affected organs is a hallmark pathological feature of IgG4-RD. However, the quantity of these cells varies by organ and may correlate with tissue size (7). It is worth noting that infiltration of IgG4+ cells has also been documented in patients with conditions other than IgG4-RD, such as rheumatoid arthritis and antineutrophil cytoplasmic antibody-associated vasculitis (34). Moreover, the serum IgG4 concentration in IgG4-RPF is low, with approximately 40-50% of patients exhibiting normal serum IgG4 levels (35). Meanwhile, the clinical presentations of IgG4-related hypertrophic pachymeningitis (IgG4-HP) resemble those of hypophysitis due to other etiologies, with serum IgG4 levels typically remaining normal, particularly in the absence of additional systemic manifestations. As a result, IgG4-RPF and IgG4-HP are infrequently identified at the onset of the disease, leading to a relatively delayed diagnosis (6, 35). Consequently, the diagnosis of IgG4-RD typically necessitates a multidisciplinary approach, integrating clinical evaluation, imaging studies, serological assessments, and histopathological analysis. In cases where diagnosis presents challenges, it is recommended to perform biopsies and pathological examinations on multiple affected regions. A diagnosis of IgG4-RD can be confirmed when a particular anatomical site exhibits distinct pathological characteristics and alternative diseases have been excluded.

The most common central nervous system (CNS) manifestations of IgG4-RD are hypertrophic pachymeningitis (HP), hypophysitis and orbital disease (29). However, involvement of the spinal dura mater is exceedingly rare. Studies have documented cases of IgG4-HSP, characterized by clinical features such as limb numbness, weakness, and symptoms associated with spinal cord compression. Furthermore, the diagnosis of IgG4-HSP is primarily dependent on dural biopsy, as no diagnostic biomarkers have been identified in cerebrospinal fluid (CSF). A study has showed that an elevated CSF IgG4 index can serve as a crucial diagnostic indicator, even in seronegative instances (36). The detection of IgG4 oligoclonal bands in CSF may suggest the blood-brain barrier impairment. In a study that utilized CSF IgG4 concentrations to differentiate IgG4-related hypertrophic pachymeningitis (IgG4-HP) from other hypertrophic pachymeningitis (OHP) disorders, CSF IgG4 levels exceeding 2.27 mg/dL successfully identified 100% of IgG4-HP cases and 5% of OHP cases. Additionally, an IgG4Loc cutoff value of 0.47 distinguished 100% of IgG4-HP cases without identifying any OHP cases (37). Nevertheless, there is presently no established consensus or guideline regarding IgG4 indicators or alternative biomarkers in the CSF of patients with IgG4-HSP. Further research is necessary to elucidate this matter in the future.

The treatment of IgG4-RD shows considerable heterogeneity. The proliferative phenotype exhibits a strong response to glucocorticoids; however, due to its high recurrence rate, maintenance therapy with immunosuppressants, such as mycophenolate mofetil, or rituximab is often required. In contrast, the fibrotic phenotype is characterized by prominent storiform fibrosis and occlusive venous inflammation, with reduced lymphocyte infiltration and a lower density of IgG4+ plasma cells. Given the irreversible nature of fibrosis and the limited response to glucocorticoids, early intensive therapeutic intervention is imperative. B-cell depletion therapy demonstrates limited efficacy in this context. Anti-fibrotic agents, such as nintedanib and pirfenidone, are generally effective in inhibiting the full activation of fibroblasts and the secretion of collagen, which have demonstrated promising effects in primary fibrotic diseases, including idiopathic pulmonary fibrosis. Given the similarities in the pathogenesis of these idiopathic fibrotic diseases and IgG4-RD, antifibrotic drugs may potentially proving effective during the fibrotic activity associated with IgG4-RD (2). In this case, tamoxifen was administered in the patient to expect an antifibrotic effect. Tamoxifen, a synthetic nonsteroidal antiestrogen commonly employed in the treatment of breast cancer, has been shown to reduce the levels of growth factors associated with fibroblast proliferation and collagen production. Some studies have substantiated its effectiveness in the treatment of retroperitoneal fibrosis and epidural fibrosis (38, 39). However, clinical experience with antifibrotic treatment for IgG4-RD remains limited, necessitating further research to elucidate its efficacy and safety. Furthermore, surgical intervention may be required to alleviate symptoms associated with compression.

Through a systematic literature review, we synthesized the clinical data from 22 reported cases of IgG4-HSP. Our analysis revealed that the majority of patients presented with isolated HSP, while only three cases (Cases No. 1, No. 5, and No. 10) demonstrated multi-organ involvement. In Case No. 1, the patient developed salivary gland inflammation, likely attributable to IgG4-RD, with a one-year interval between the onset of this condition and the emergence of HSP symptoms. In Case No. 5, the patient concurrently presented with cavernous sinus syndrome, right retro-orbital headache, and HSP. In Case No. 10, however, there is no documentation regarding the duration from the initial presentation of sialisterium to the onset of HSP. In the case presented in this report, the interval between the development of RPF and the occurrence of HSP was 6 months. Notably, the serum IgG4 positivity rate in these patients was less than 50%. Among the 19 cases with documented serum IgG4 levels, 8 exhibited elevated IgG4 levels, with a mean age of 58 years, comprising 3 males. Conversely, 9 cases demonstrated normal IgG4 levels, with a mean age of 51 years, including 5 males. Comparative analysis between the two groups revealed no statistically significant differences in age (t = 0.8725, p = 0.3967) or gender (χ² = 0.5542, p = 0.6372). Due to the wide time span of the published literature and inconsistent recording standards, a substantial portion of the patients’ biopsy pathological data is incomplete. For instance, the status of storiform fibrosis or obliterative phlebitis was documented in only 7 patients. Consequently, conducting further subgroup comparative analyses of the existing pathological data is currently unfeasible.

Upon the 22 cases, first-line treatment universally comprised surgical decompression and glucocorticoids, with variations in the use of adjunctive immunosuppressive agents. Although most patients showed varying degrees of improvement after treatment, there were still some patients who experienced recurrence or death. In detail, two cases (Cases No. 4 and No. 8) resulted in mortality due to severe infection. In Case No. 4, the patient underwent decompression surgery and was administered one gram of pulse intravenous methylprednisolone daily for three days during hospitalization. Upon discharge, the patient was transitioned to 60 mg of oral prednisone. Due to an incomplete therapeutic response, second-line treatment with rituximab was initiated. Unfortunately, despite initial clinical improvement, the patient succumbed to infection secondary to immunosuppression 4.5 months after presentation. In Case No. 8, the patient also underwent decompression surgery and received GC and CTX therapy (specific dosages and duration are not provided in this report). However, the patient developed pulmonary fungal infection 4 months later. Despite receiving standard anti-infective treatments, the patient ultimately died 9 months after diagnosis. Besides, there cases (No.10, No.13 and No.19) experienced disease recurrence. In case No.10, the patient received decompression surgery, GC and MTX and recurred 2 years later, but the dosages and duration of drugs and the reason for recurrence were not mentioned. In case No.13, the patient was first underwent 6 weeks of physical therapy and received three cycles of epidural steroid injection with no relief, and then she underwent decompression surgery, without any other therapy. 1 year later, the patient experienced a recurrence and underwent another surgical treatment. She was prescribed 60 mg of prednisone daily and completed a two-month course of steroid dose reduction therapy. At her most recent follow-up, she had complete recovery and no recurrence. In Case No. 19, the patient underwent decompression surgery and was administered a moderate dose of GC (specific dosages and duration are not provided in this report), which elicited a favorable response. However, 4 years later, the patient recurred upon reducing the prednisone dosage to 4 mg per day for maintenance, so the treatment regimen was adjusted to include a higher dose of prednisone in conjunction with anti-rheumatic drugs (specific medications were not detailed in the report), resulting in an alleviation of the patient’s condition.

In the presented case, the female patient initially presented with symptoms indicative of urinary tract obstruction. Abdominal CT imaging revealed diffuse soft tissue density within the retroperitoneum, and a percutaneous biopsy confirmed the presence of RPF. The diagnosis of IgG4-RD was not established initially due to normal serum IgG4 levels and sparse IgG4+ plasma cells (<10/HPF) upon immunohistochemical analysis of the retroperitoneal mass. However, when reviewing the biopsy results of retroperitoneal mass, we can still observe some changes in the pattern of storiform fibrosis of tissue and the infiltration of lymphocytes, eosinophils and plasma cells in the HE staining of pathological sections. Histopathological examination of the spinal lesion, guided by the 2020 revised comprehensive diagnostic criteria for IgG4-RD, led to a diagnosis of probable IgG4-RD. This diagnosis was based on characteristic lymphoplasmacytic infiltration, storiform fibrosis, and the presence of more than 10 IgG4+ plasma cells per HPF in only one focus. Combined with clinical manifestation and histopathological results, we thus conclude that both RPF and HSP in this patient represent distinct clinical manifestations of IgG4-RD. A noteworthy aspect of this case is the patient’s normal serum IgG4 levels and histopathological findings characterized by prominent storiform fibrosis, with IgG4+ plasma cell counts barely reaching the diagnostic threshold for IgG4-RD. The patient initially presented with RPF, followed by the development of HSP, with a prolonged disease course. During the disease progression, rituximab therapy was implemented, effectively suppressing B cell production. As a result, the current pathological findings are primarily characterized by fibrosis, which accounts for the reduced presence of IgG4+ plasma cells observed in the patient’s dural pathology. Moreover, our literature review indicates that IgG4-HSP is predominantly reported as an isolated condition, with concurrent RPF being rarely documented in the existing medical literature.

A review of existing literature on HSP indicates that nearly all patients have undergone spinal decompression surgery, underscoring the importance of timely decompression for improving prognosis once symptoms of spinal cord compression appear in HSP patients. In this particular case, the patient exhibited a critically compromised spinal cord status upon admission. Immediate transfer to the neurosurgery department for surgical decompression, followed by postoperative administration of glucocorticoids and immunosuppressive therapy, led to favorable clinical outcomes. Consequently, a multimodal therapeutic approach—comprising surgical decompression, glucocorticoids, antifibrotic agents, and immunosuppressants—resulted in significant clinical and radiological improvement. But which immunosuppressants would be suitable for patients with IgG4-HSP combined with RPF as preferred choice needs more clinical evidence and trails.

## Limitations

This article acknowledges several limitations. Firstly, due to the critically compromised status of the spinal cord, a lumbar puncture was contraindicated in this patient, preventing the assessment of IgG4 levels in the cerebrospinal fluid. Secondly, the rarity of the disease resulted in a small sample size of included cases, which precluded systematic statistical analyses. Thirdly, the case reports analyzed demonstrated heterogeneity in their sources, leading to incomplete or missing critical clinical data.

## Conclusion

In conclusion, we present a rare case of IgG4-HSP combined with RPF, emphasizing the unique aspects of its clinical diagnosis and management, particularly the decision-making process in diagnostic and therapeutic strategies. We are encouraged by the favorable prognosis observed in this patient and will continue to monitor her therapeutic outcomes through long-term follow-up.

We recommend that clinicians advise patients with RPF who exhibit symptoms or signs of myelopathy to seek immediate consultation with a neurology department. Early multidisciplinary collaboration, encompassing pathology, imaging, and clinical evaluation, along with long-term immunosuppression, is crucial for effective management.

## Data Availability

The original contributions presented in the study are included in the article/supplementary material. Further inquiries can be directed to the corresponding authors.
